# Tertiary butylhydroquinone alleviated liver steatosis and increased cell survival via β-arrestin-2/PI3K/AKT pathway

**DOI:** 10.22038/IJBMS.2021.58156.12924

**Published:** 2021-10

**Authors:** Tian-tian Zhu, Chao-nan Zhu, Yue Qiu, Qian-Shuai Li, Xin Yu, Guo-Jie Hao, Ping Song, Jian Xu, Peng Li, Ya-ling Yin

**Affiliations:** 1 College of Pharmacy, Xinxiang Medical University, Xinxiang, China; 2 Xinxiang Key Laboratory of Vascular Remodeling Intervention and Molecular Targeted Therapy Drug Development, Xinxiang, China; 3 Department of Pharmacy, The first Affiliated Hospital of Xinxiang Medical University, Xinxiang, China; 4 Henan International Joint Laboratory of Cardiovascular Remodeling and Drug Intervention, Xinxiang, China; 5 School of Basic Medical Sciences, Xinxiang Medical University, Xinxiang, China, 453003

**Keywords:** Insulin resistance, Liver steatosis, Tertiary butylhydroquinone, Type 2 diabetes mellitus, β-arrestin-2

## Abstract

**Objective(s):**

This study aimed to evaluate the effects and the underlying mechanisms of tertiary butylhydroquinone (TBHQ) on diabetic liver steatosis and cell survival.

**Materials and Methods:**

We performed streptozocin injection and used a high-sugar-high-fat diet for mice to develop an animal model of type 2 diabetes mellitus (T2DM). Bodyweight, blood glucose levels, and content of insulin were measured on all of the mice. The liver tissues were observed by hematoxylin-eosin staining. Protein levels of the liver were measured by Western blot analysis in mice. Primary hepatocytes were induced by hypochlorous acid (HClO) and insulin to form insulin resistance (IR). Primary hepatocyte apoptosis was observed by Hoechst staining. The PI3K/AKT signaling pathway and β-arrestin-2 factor were evaluated by Western blot assay.

**Results:**

TBHQ reduced the blood glucose level and content of insulin in serum, increased body weight, and effectively alleviated liver steatosis in diabetic mice. TBHQ significantly up-regulated the expression of p-PI3K, p-AKT, GLUT4, GSK3β, and β-arrestin-2 in the liver of diabetic mice. Cell experiments confirmed that TBHQ increased the survival ability of primary hepatocytes, and TBHQ improved the expression of p-PI3K, p-AKT, GLUT4, and GSK3β by activating β-arrestin-2 in primary hepatocytes.

**Conclusion:**

TBHQ could alleviate liver steatosis and increase cell survival, and the mechanism is due in part to β-arrestin-2 activation.

## Introduction

Insulin resistance (IR) is an important cause for the development of type 2 diabetes mellitus (T2DM) (1-4), which is a disease associated with abnormal metabolic function and will lead to abnormally high levels of blood glucose (5, 6). The long-term existence of hyperglycemia in diabetes leads to liver steatosis. Therefore, ameliorated IR plays a central role in treating liver steatosis of T2DM.

Tertiary butylhydroquinone (TBHQ) is the most commonly used synthetic antioxidant and can enhance the effect of insulin *in vivo* (7, 8). TBHQ remarkably increases PI3-kinase-dependent Akt (also referred to as PKB) phosphorylation (p-Akt) (9, 10), which could reflect insulin sensitivity in hepatocytes and nerve cells (11, 12). The PI3K/Akt signaling pathway plays a vital role in regulating cell apoptosis and glucose metabolism (13). The activation of p110 and p85 subunits of PI3K can catalyze Akt phosphorylation, then lead to positive regulation of the PI3K/Akt signaling pathway (14, 15). In addition, p-Akt stimulates the glucose metabolism via controlling the insulin-stimulated translocation of GLUT4 that plays an important role in insulin action (16) and controlling the activity of glycogen synthesis kinase-3β (GSK-3β) that has an important effect on glycogen synthase, thereby promoting glucose uptake, glycogen synthesis, insulin sensitivity (17, 18) and regulation of the cell cycle (19, 20). However, the underlying mechanism of TBHQ increases p-Akt thus enhances insulin effect, and hepatocytes apoptosis is unclear.

The β-arrestin family has two ARRBs isoforms, ARRB1 and ARRB2, which share a high degree of structural homology (21, 22). β-arrestin functions as molecular scaffold for a myriad of signal proteins (23). Some studies demonstrate that hepatic β-arrestin-2, a key cellular regulator with multifunctional roles, plays a vital role in maintaining euglycemia by acting as an effective negative regulator of hepatic GCGR signaling *in vivo* (24, 25). Accumulating evidence demonstrates that β-arrestin-2 mediated pathways participate in regulating insulin sensitivity (26). β-arrestin-2 regulates insulin action through a complex containing insulin receptor, c-Src, and Akt/PKB. The complex allows c-Src to phosphorylate Tyr315 and 326 residues of Akt, which are necessary for subsequent phosphorylation of Akt on thr308 and ser473 by PDK1 and PDK2, separately (27). β-arrestin-2 and AMPKα2 are functionally consistent (28-34), TBHQ can activate AMPKα2 (35). Therefore, whether TBHQ can improve IR through β-arrestin-2 remains to be verified.

The aim of this study, therefore, was to establish a link between β-arrestin-2 and PI3K/Akt signaling pathway-related hepatocyte apoptosis and liver steatosis, then to determine if TBHQ could inhibit these pathological processes by activating the pathway. The work described here demonstrates that exposure of hepatocytes to TBHQ increased β-arrestin-2 expression, promoted PI3K/AKT pathway, blocked hepatocytes apoptosiss, and liver steatosis.

## Materials and Methods

***Materials and reagents***

Tertiary butylhydroquinone (TBHQ, IT1150, Soleibao, Beijing, China), Streptozocin (STZ, A610130, Sangon, Shanghai, China), Sodium hypochlorite solution (NaClO, A501944, Sangon, Shanghai, China). Insulin (I-5500, Sigma, MO, USA). Fetal bovine serum (FBS) and Dulbecco’s modified eagle’s medium (DMEM) were obtained from Gibco BRL (Carlsbad, CA, USA). Hematoxylin-eosin (HE) staining kit (C0105), Hoechst staining kit (C0003), and bicinchoninic acid (BCA) protein assay kit (P0012) were purchased from Beyotime Biotechnology Research Institute (Shanghai, China). Primary antibodies against PI3K (1:500, D162051, Sangon, Shanghai, China), p-PI3K (1:1000, #17366, Cell Signaling Technology), AKT (1:500, D199241, Sangon, Shanghai, China), p-AKT (1:1000, ab38449, Abcam, USA), β-arrestin-2 (1:1000, ab54790, Abcam, USA), GSK3β (1:1000, ab32391, Abcam, USA), GLUT4 (1:1000, ab654, Abcam, USA), β-actin (1:1000, AF0003, Beyotime, Shanghai, China) were used. ARRB2 siRNAs were acquired from the riboFECT ™ CP transfection kit (Guangzhou, China).

***Protocols for animal experiments***

Four-week-old male ApoE-/-mice were purchased from Model Animal Research Center GemPharmatech Co., Ltd. of Nanjing University (Qualified number: 201804855). These animal studies were approved by the Xinxiang Medical University Veterinary Medicine Animal Care and Use Committee. The mice were housed in a temperature and humidity-controlled room on a light/dark cycle of 12 hr. Low-dose of STZ and high-sucrose-high-fat diet has been used to induce the mice model of T2DM.

The mice were randomly divided into 5 groups (n=10 for each group) after one week of acclimation period: The control group, mice fed a conventional diet; The control+TBHQ group, mice fed a conventional diet and gavaged with TBHQ at a dose of 60 mg/kg/day(36, 37); The T2DM group, mice fed a high-sucrose-high-fat diet; The T2DM+TBHQ group, mice fed a high-sucrose-high-fat diet and gavaged with TBHQ at a dose of 60 mg/kg/day; The T2DM+rosiglitazone group, mice fed a high-sucrose-high-fat diet and gavaged with rosiglitazone at a dose of 7 mg/kg/day (38, 39). The T2DM group, the T2DM+TBHQ group, and the T2DM+rosiglitazone group of mice were subjected to an injection of STZ (50 mg/kg body weight in 10 mM citrate buffer, pH 4.0, IP) after a 10 hr fast (40) and one week later, the T2DM+TBHQ group and the T2DM+rosiglitazone group began receiving a high-sucrose-high-fat diet and were gavaged with TBHQ and rosiglitazone.

**Figure 1:**
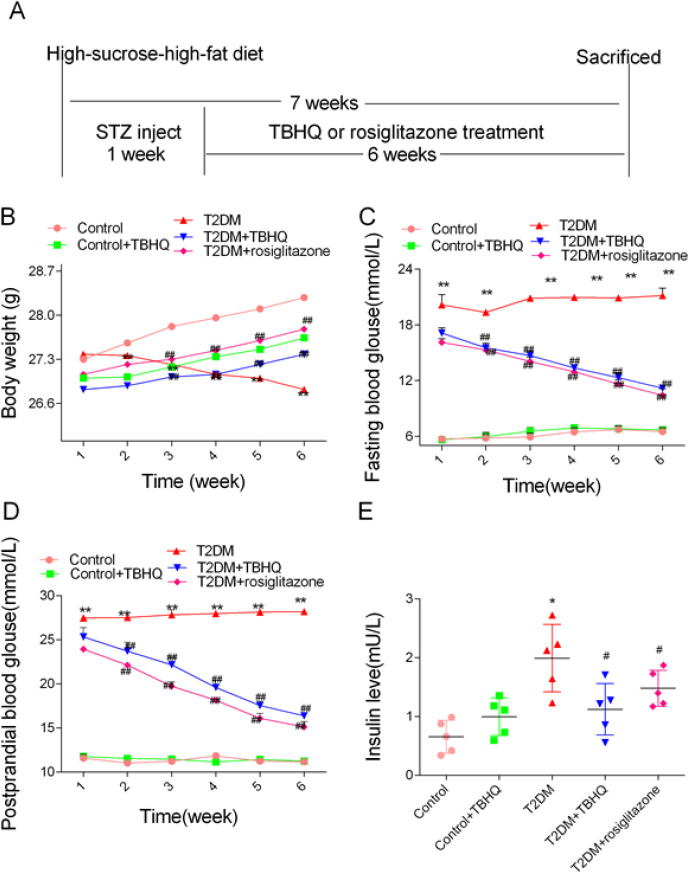
Effects of TBHQ treatment on fasting blood glucose, postprandial blood glucose, body weight, and insulin levels of serum. A, protocol of animal experiment; B, bodyweight; C, fasting blood glucose; D, postprandial blood glucose; E, insulin levels of serum. TBHQ stands for tert-butyl hydroquinone 60 mg/kg. **P<0.01 vs Control; ##*P*<0.01 vs T2DM. Results are represented as mean±SEM (n=10)

All mice were given a high-sucrose-high-fat diet (sucrose: lard: sodium cholate: cholesterol: conventional feed=20: 10: 7: 3: 60) except for the control group and control+TBHQ group during the entire treatment period (41). The protocol of the animal experiment was shown in Figure 1A. 

***Cell culture and treatments***

The *in vitro* experiment was done with primary hepatocytes from mice. Primary hepatocytes were cultured in DMEM containing 25 mM glucose, 10% FBS, 100 U/ml penicillin, and 100 μg/ml streptomycin in a humidified incubator containing 5% CO_2_ at 37 °C. To induce an IR model, primary hepatocytes were exposed to HClO (200 mM) for 40 min and then insulin for 25 min. The experimental groups were as follows: (1) Control group, (2) TBHQ group, (3) HClO group, (4) Insulin group, (5) HClO+TBHQ group, (6) HClO+insulin group, (7) TBHQ+insulin group, and (8) HClO+TBHQ+insulin group.

***Biochemical study***

The body weight and blood glucose levels of all mice were measured each week during the experimental period. Blood was collected from the mice eye socket vein and centrifuged at 4000 rpm for 15 min at 4 °C to isolate serum. Serum and liver samples were stored in a -80 °C refrigerator for further biochemical assessment (42). All animals’ liver tissues were collected.

***Assay of serum insulin***

Serum insulin levels of mice were assayed using an ELISA kit following the manufacturer’s instructions.

***Hematoxylin-eosin (HE) staining***

Liver tissues fixed in 4% PBS-buffered neutral formaldehyde for 48 hr were embedded in paraffin and cut into a section with 4 μm thickness, then stained by HE staining (43). Lesion area related to swollen hepatocytes, granular degeneration, steatosis, and lipid vacuolization in the liver, was contrast-adjusted with the Image J software (National Institutes of Health, Bethesda, MD, USA).

***Apoptosis experiments***

According to the manufacturer’s instructions, Hoechst staining was performed using the Hoechst staining kit in primary hepatocytes. The apoptosis cells were counted using the Image J software (National Institutes of Health, Bethesda, MD, USA).

***Western blot analysis***

Protein levels were analyzed by Western blot analysis (44, 45). Primary antibodies against PI3K (1:500, D162051, Sangon, Shanghai, China), p-PI3K (1:1000, #17366, Cell Signaling Technology), AKT (1:500, D199241, Sangon, Shanghai, China), p-AKT (1:1000, ab38449, Abcam, USA), β-arrestin-2 (1:1000, ab54790, Abcam, USA), GSK3β (1:1000, ab32391, Abcam, USA), GLUT4 (1:1000, ab654, Abcam, USA), β-actin (1:1000, AF0003, Beyotime, Shanghai, China) were used in this study.

***Cell transfection***

β-arrestin-2 was transfected by using the RibobioFECT TM CP transfection kit. β-arrestin-2 siRNA1 (50 nM) was selected for further experiments by quantitative real-time polymerase chain reaction (qRT-PCR), Western blot, and immunofluorescence (46).

***Statistical analysis***

Statistical analysis was performed using SPSS software. All quantitative results data were expressed as mean±SEM. All results were performed by one-way ANOVA with Newman-Student-Keuls test for multiple comparisons, and for both sides *P*<0.05 were considered significant.

## Results

***TBHQ obviously alleviated the abnormal glucose metabolism in diabetic mice***

A high-sucrose-high-fat diet treatment and STZ injection-induced diabetes. The fasting blood glucose (FBG), postprandial blood glucose (PBG), and bodyweight levels of experimental mice are indices of diabetes. As shown in Figure 1B-D, data were examined over different time intervals of all experimental mice. FBG and PBG significantly decreased in comparison with diabetic mice after six weeks of rosiglitazone or TBHQ treatment. The bodyweight of the T2DM group obviously decreased during six weeks. However, the bodyweight of the T2DM group significantly increased with TBHQ or rosiglitazone treatment. We examined the serum insulin levels of all experimental groups, as shown in Figure 1E. IR was observed in the T2DM group as evident by increasing serum insulin levels compared with the control group. The serum insulin values obviously decreased in comparison with diabetic mice after six weeks of rosiglitazone or TBHQ treatment. These data demonstrate that the persistent application of TBHQ relieves glucose metabolic disorders and IR in diabetic mice induced by high-sucrose-high-fat diet treatment and STZ injection.

***TBHQ effectively alleviated liver steatosis of diabetic mice and increased cell survival in primary hepatocytes***

**Figure 2:**
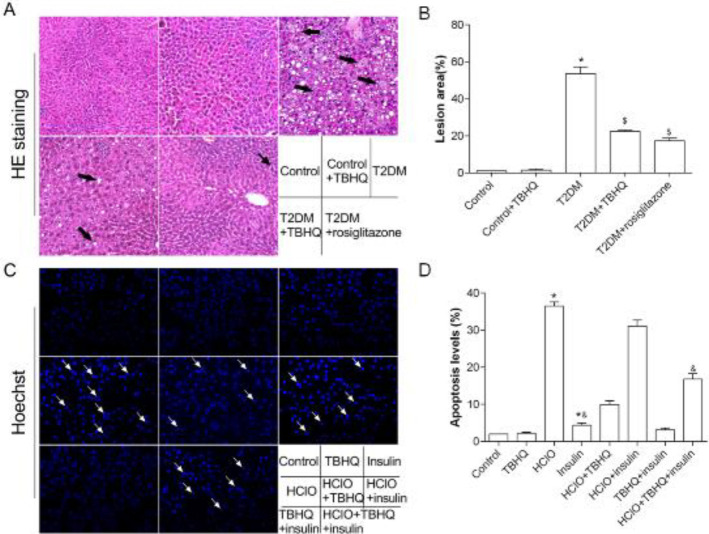
Effects of TBHQ on liver steatosis of diabetic mice and cell survival in primary hepatocytes. A, hepatic injury included swollen hepatocytes, granular degeneration, steatosis, and lipid vacuolization in diabetic mice. HE staining of liver sections, original magnification ×200; B, lesion area analysis of HE staining; C, cell survival in primary hepatocytes. Hoechst staining of primary hepatocytes, original magnification ×200; D, apoptosis level analyses of hepatocytes. *P<0.05 vs Control, $*P*<0.05 vs T2DM. &*P*<0.05 vs HClO+insulin. Results are represented as mean±SEM (n=10)

TBHQ: tertiary butylhydroquinone; HE: HCLO: hematoxylin-eosin; hypochlorous acid

To directly assess whether TBHQ treatment has influences on T2DM, the lesion area in the liver was determined by HE staining (Figure 2A). As indicated in Figures 2A-B, Liver histopathological examination showed no abnormality of the hepatocyte architecture and morphology in normal mice. In contrast, high-sucrose-high-fat diet treatment and STZ injection-induced T2DM mice exhibited severe hepatocyte necrosis and macrovesicular steatosis in the hepatocytes. Whereas TBHQ feeding for six weeks exhibited a beneficial effect on diabetes-mediated pathological changes, the degeneration of the hepatocytes markedly alleviated, and macrovesicular steatosis and cell death partially recovered. These results demonstrate that the persistent application of TBHQ relieves hepatic function in diabetic mice.

Hoechst staining was used to explore the effect of TBHQ on primary hepatocyte apoptosis. The cells with apoptotic morphology appearing condensed or fragmented nuclei were counted. As shown in Figures 2C-D, cell treatment with HClO, TBHQ, and insulin displayed varying degrees of apoptosis, we found that HClO promotes cells apoptosis. The number of apoptosis cells markedly down-regulated after TBHQ treatment. These results indicate that TBHQ subsequently increases the survival ability of primary hepatocytes.

***Effects of TBHQ treatment on the PI3K/AKT pathway in diabetic mice and primary hepatocytes of insulin resistance***

**Figure 3:**
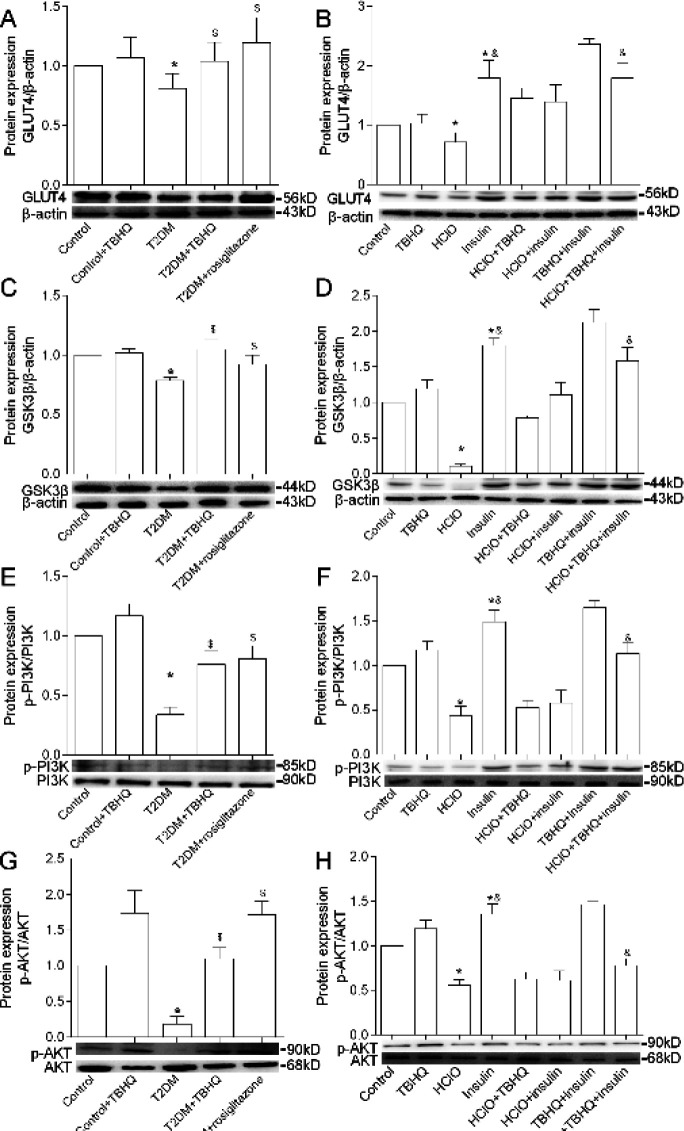
Protein expression of levels p-PI3K, p-AKT, GLUT4, and GSK3β in the liver of mice and primary hepatocytes. p-PI3K, PI3K, p-AKT, AKT, GLUT4, GSK3β, and β-actin stand for phospho-PI3K-p85-alpha-tyr607-antibody-polyclonal, anti-PI3K-p85 alpha rabbit pAb, phospho-AKT-S473 rabbit pAb, AKT polyclonal antibody, GLUT4 polyclonal antibody, GSK3β polyclonal antibody, and β-actin rabbit polyclonal antibody, respectively. *P<0.05 vs Control, $*P*<0.05 vs T2DM, &*P*<0.05 vs HClO+insulin. Results are represented as mean±SEM (n=3)

PI3K: phosphatidylinositol 3-kinase; AKT: protein kinase B; GLUT4 glucose transport-4; GSK3β: glycogen synthase kinase-3β

To confirm the regulatory effect of TBHQ on liver tissues, we next evaluated the glucose metabolism-related proteins. The hepatic protein levels of GLUT4, GSK3β, p-PI3K, and p-AKT are the indices of diabetes (Figures 3A, C, E, and G). We found that TBHQ increased protein expression of the glucose transport marker GLUT4 and glycogen synthase marker GSK3β when compared with the T2DM group. Moreover, the p-PI3K and p-AKT levels were relatively elevated in diabetic mice in comparison with T2DM mice. Many studies have shown that the protein expression level of p-AKT is up-regulated by the phosphorylation of PI3K (47). Therefore, the level of p-AKT in liver tissues was inspected to discuss whether TBHQ increased p-AKT activity by activating the PI3K pathway and then regulating gluconeogenesis. These results suggest that TBHQ may inhibit glucose uptake and increase glycogen synthesis via the PI3K/AKT pathway.

To further confirm the regulatory effect of TBHQ on suppressing glucose uptake and increase glycogen synthesis in the liver of diabetic mice via the PI3K/AKT pathway, we conducted a cell experiment and found that HClO and insulin treatment decreased protein levels of GLUT4, GSK3β, p-AKT, and p-PI3K in primary hepatocytes, which were effectively improved by TBHQ in comparison with cells of insulin group (Figures 3B, D, F, H). Exposure of primary hepatocytes to TBHQ significantly enhanced the phosphorylation levels of AKT and PI3K in HClO and insulin-treated primary hepatocytes. Protein levels of p-PI3K, p-AKT, GLUT4, and GSK3β were relatively elevated in comparison with HClO and insulin-treated primary hepatocytes. Collectively, these results further demonstrate that TBHQ attenuates diabetes by inhibiting glucose uptake and increasing glycogen synthesis via the PI3K/AKT pathway.

***TBHQ might activate*** ***β*** ***-*** ***arrestin-2*** ***factor in diabetic mice and primary hepatocyte cells of insulin resistance***

**Figure 4:**
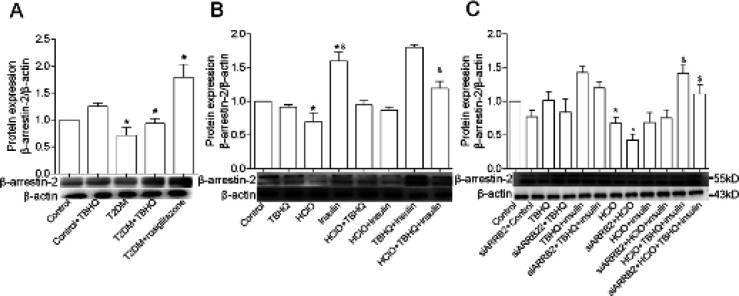
Effect of TBHQ on the expression of β-arrestin-2. TBHQ, β-arrestin-2, and β-actin stand for tert-butylhydroquinone 60 mg/kg, beta-arrestin-2 rabbit polyclonal antibody, and β-actin rabbit polyclonal antibody, respectively. *P<0.05 vs Control, #*P*<0.05 vs T2DM, &*P*<0.05 vs HClO+insulin, ^*P*<0.05 vs siARRB2+Control, $*P*<0.05 vs siARRB2+HClO+insulin. Results are represented as mean±SEM (n=3)

TBHQ: tertiary butylhydroquinone; HCLO: hematoxylin-eosin; hypochlorous acid

To examine whether β-arrestin-2 activation is necessary for TBHQ to ameliorate glucose metabolism, we detected β-arrestin-2 in our animal and cell experiments and found that the expression of β-arrestin-2 has significantly decreased in both diabetic mice liver and IR cells (Figures 4A-B). However, after TBHQ treatment, β-arrestin-2 was signiﬁcantly increased, which has the same effect when compared with rosiglitazone. TBHQ can correct the abnormal glucose metabolism and increase the expression of β-arrestin-2, but whether TBHQ improves glucose metabolism via β-arrestin-2 still needs to be illuminated. We reduced the expression of β-arrestin-2 with β-arrestin-2-targeting siRNA in primary hepatocytes. Then we found that after TBHQ treatment, β-arrestin-2 in HClO+insulin-treated primary hepatocytes was relatively elevated in comparison with β-arrestin-2 down-regulated HClO+insulin-treated primary hepatocytes (Figure 4C). Collectively, these data suggest that TBHQ can improve IR by activating the β-arrestin-2 factor.

***TBHQ activated β-arrestin-2 factor to increase insulin sensitivity in HClO and insulin-treated primary hepatocytes***

**Figure 5:**
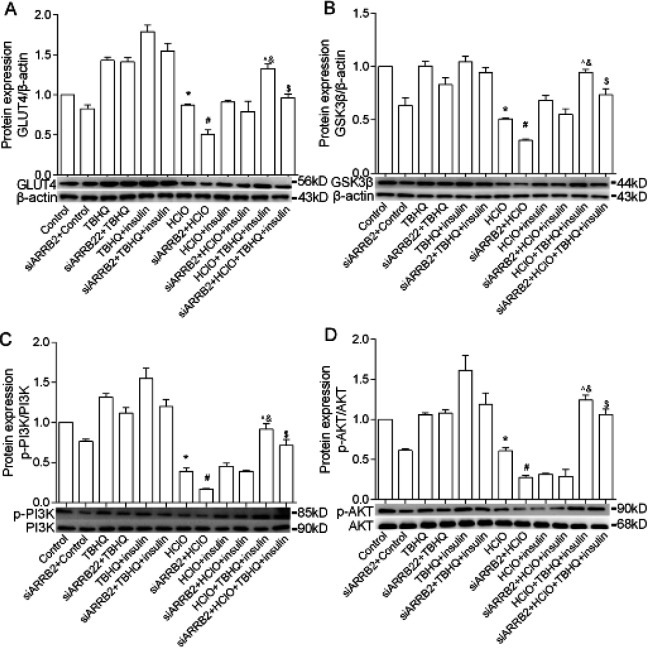
Effect of β-arrestin-2 knockdown on TBHQ treatment insulin resistance of primary hepatocytes. TBHQ, p-PI3K, PI3K, p-AKT, AKT, GLUT4, GSK3β, and β-actin stand for tert-butylhydroquinone 60 mg/kg, phospho-PI3K-p85-alpha-tyr607-antibody-polyclonal, anti-PI3K-p85 alpha rabbit pAb, phospho-AKT-S473 rabbit pAb, AKT polyclonal antibody, GLUT4 polyclonal antibody, GSK3β polyclonal antibody, and β-actin rabbit polyclonal antibody, respectively. *P<0.05 vs Control, &*P*<0.05 vs HClO+insulin, #*P*<0.05 vs siARRB2+Control, $*P*<0.05 vs siARRB2+HClO+insulin, ^*P*<0.05 vs siARRB2+HClO+TBHQ+insulin. Results are represented as mean±SEM (n=3)

TBHQ: tertiary butylhydroquinone; PI3K: phosphatidylinositol 3-kinase; AKT: protein kinase B; GLUT4: glucose transport-4; GSK3β: glycogen synthase kinase-3β; HCLO: hematoxylin-eosin; hypochlorous acid

To validate our hypothesis that TBHQ relieves IR by activating the β-arrestin-2 factor, we used β-arrestin-2 siRNA to decrease β-arrestin-2 expression in primary hepatocytes. Then protein expression levels of p-AKT, p-PI3K, GLUT4, and GSK3β were detected. The protein expression levels of p-AKT, p-PI3K, GLUT4, and GSK3β enhanced in TBHQ-treated primary hepatocytes in comparison with HClO and insulin-treated primary hepatocytes, whereas, blocking of β-arrestin-2 markedly retarded TBHQ-induced up-regulation of p-AKT, p-PI3K, GLUT4, and GSK3β (Figures 5A-D). Taken together, these results suggest that TBHQ activates the β-arrestin-2 factor to improve the protein expression of GLUT4, GSK3β, p-PI3K, and p-AKT in case of insulin resistance.

TBHQ: tertiary butylhydroquinon0065

**Figure 6:**
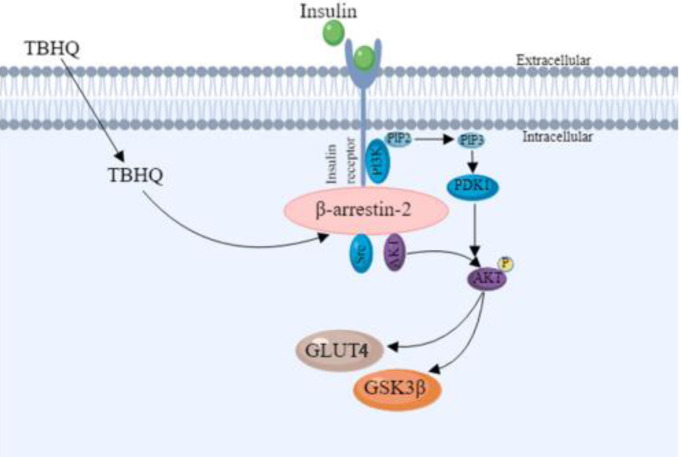
Schematic diagram showing that TBHQ alleviates the T2DM via β-arrestin-2/AKT/PI3K pathway. During T2DM, TBHQ activates β-arrestin-2, then β-arrestin-2 promotes phosphorylation of phosphatidylinositol 3-kinase subunit p85 and AKT. p-AKT reacts with glucose transporter GLUT4 and glycogen synthesis kinase GSK3β in liver tissue, eventually alleviating T2DM

TBHQ: tertiary butylhydroquinone; T2DM: type 2 diabetes mellitus; PI3K: phosphatidylinositol 3-kinase; AKT: protein kinase B; GSK3β: glycogen synthase kinase-3β

## Discussion

Insulin-resistant induced liver steatosis and hepatocellular injury (48) are complications of T2DM (1-6). TBHQ, as a food antioxidant, can increase the effect of insulin, thus reducing IR via p-Akt (7-10), which plays a vital role in regulating glucose metabolism and cell apoptosis (13). However, whether TBHQ can alleviate liver steatosis and the mechanisms behind the anti-insulin resistance and anti-apoptosis effects of TBHQ has not been clearly defined. Our data presented here was the first to show the effectiveness of TBHQ on improving liver steatosis in T2DM and the mechanism of the anti-insulin resistance and anti-apoptosis effects of TBHQ related to the β-arrestin-2/PI3K/AKT pathway. The data suggested that the persistent application of TBHQ could effectively reduce the levels of blood glucose, increase the body weight, alleviate liver steatosis and activate β-arrestin-2. Also, a parallel experiment in cells was conducted. *In vitro* studies suggested that TBHQ could increase the survival of HClO induced insulin-resistant primary hepatocytes and the mechanism related to the β-arrestin-2/PI3K/AKT pathway. In brief, TBHQ may potentially be used as a medicinal compound for the treatment of T2DM via activating the β-arrestin-2/PI3K/AKT pathway.

TBHQ, which has antioxidant and anti-inflammatory effects, can protect pancreatic islet cells from damage and increase insulin sensitivity *in vivo* to improve T2DM. Liver tissue, as a main detoxifying organ, is often damaged by metabolic overload, lipid accumulation, and viruses’ factors. Moreover, liver tissue metabolic disturbance will occur in T2DM. TBHQ can inhibit liver cell apoptosis, which protects the liver from acute and chronic toxin-mediated injury (35, 49, 50). According to these pieces of evidence, we hypothesized that TBHQ can alleviate liver injury in T2DM, and our subsequent experiment supports this point.

β-Arrestin-2 is a key component in the regulation of insulin sensitivity (15), and the lack of β-arrestin-2 can inhibit the resistibility of insulin in glucose uptake, which leads to abnormal glucose tolerance *in vivo* (51). TBHQ can activate β-arrestin-2 and promote the autophagy of hepatocytes, thus exerting the effect of anti-fatty acid (25). In our experiments, we detected the expression of β-arrestin-2 both in *in vivo* and *in vitro* studies. Consistently, we found that β-arrestin-2 expression has decreased in both diabetic mice liver and IR cells. However, after TBHQ treatment, the expression of β-arrestin-2 has significantly increased. In order to further explore the underlying mechanism, we used β-arrestin-2 siRNA in primary hepatocytes and found that β-arrestin-2 in HClO+insulin-treated primary hepatocytes was relatively elevated in comparison with β-arrestin-2 down-regulated HClO+insulin-treated primary hepatocytes after TBHQ treatment, which suggested that TBHQ alleviated insulin-resistant directly via β-arrestin-2. Previous reports have suggested that insulin can tremendously decrease blood glucose by improving phosphorylates AKT and PI3K, which is associated with GSK3β and GLUT4 (52-54). β-arrestin-2 can induce AKT activation by phosphorylating two major residues: Ser473 and Thr308 (25). Thus, we guessed that TBHQ alleviates IR and liver steatosis via activating β-arrestin-2/PI3K/AKT pathway. Our results presented that TBHQ could enhance the phosphorylation levels of AKT and PI3K, thus significantly improving the role of GLUT4 and GSK3β in the liver of diabetic mice and primary hepatocytes of IR. After β-arrestin-2 knockdown, the PI3K/AKT pathway was significantly influenced.

Rosiglitazone, a thiazolidinedione antidiabetic agent, improves IR (55). In animal models of IR, rosiglitazone decreased plasma glucose, insulin, and triglyceride levels and also attenuated or prevented diabetic nephropathy and pancreatic islet cell degeneration (55). Some studies suggested that rosiglitazone promotes glucose metabolism of GIFT tilapia based on the PI3K/Akt signaling pathway (56), which may have the same effect as TBHQ in treating T2DM. Clinical analysis has suggested that it should be used with caution in patients with cardiac insufficiency, severe cardiovascular disease, and hypertension (57). It also may increase the occurrence of cardiovascular complications in the treatment of T2DM. Therefore, developing high-efficiency and safe drugs that can prevent diabetes is very necessary. TBHQ is mainly used as a food additive. TBHQ is safe and low toxicity for the human body, and its chemical structure is modified to enhance the antioxidant effect. In our study, we found that TBHQ can improve IR except for the antioxidant effect . Thus TBHQ is expected to be the ideal medicine for treating T2DM.

As we all know, diabetes can cause damage to the liver, skeletal muscles, and fat. The shortcoming of this study is that we have only explored the effect of TBHQ in relieving liver damage, we have not probed the role of TBHQ in improving injury of skeletal muscles and fat. In the next study, we will use C2C12 and 3T3-L1 cells to research the effect of TBHQ in these diseased organs. As different tissues have different sensitivities to TBHQ, we will use multiple doses of TBHQ to explore the optimal dose of TBHQ in diabetes. Only in this way can we make TBHQ an ideal medicine and use it as soon as possible for treating T2DM patients.

## Conclusion

In summary, this study demonstrates that TBHQ alleviates T2DM via β-arrestin-2 activation and β-arrestin-2 mediates liver steatosis and cell apoptosis via PI3K/AKT signaling pathway (Figure 6). Therefore, TBHQ can be a potential medicine and β-arrestin-2 is a pharmacological target for the therapy of T2DM.

## Availability of Data and Material

The data supporting the findings can be found in supplementary materials.

## Cod Availability

Not applicable.

## Ethics Approval

All experimental protocols were administrated in strict accordance with the Guidance of the Laboratory Animal Center of Henan Province, Xinxiang Medical University, China.

## Consent to Participate

All authors were involved in drafting this work for important intellectual content, and are accountable for all aspects presented in this study.

## Consent for Publication

All authors agreed to the version published, and are accountable for all aspects presented in this study.

## Authors’ Contributions

SP, XJ, LP, and YYL Study conception and design; ZCN and QY Data analyzing and draft manuscript preparation; ZTT Critical revision of the paper; LTH, HGJ, and YYF Supervision of the research; ZTT, ZCN, QY, YYF, HGJ, LTH, SP, XJ, LP, YYL Final approval of the version to be published.

## Conflicts of Interest

The authors have no conflicts of interest to declare.
